# The role of multimorbidity and socio-economic characteristics as potential risk factors for Long Covid: evidence from the multilevel analysis of the Survey of Health, Ageing and Retirement in Europe’s corona surveys (2020–2021)

**DOI:** 10.1093/ageing/afad225

**Published:** 2023-12-19

**Authors:** Piotr Wilk, Valerie Moran, Maria N Pi Alperin, Torsten Bohn, Guy Fagherazzi, Maurice P Zeegers, Maria Ruiz-Castell

**Affiliations:** Department of Epidemiology and Biostatistics, Schulich School of Medicine & Dentistry, Western University, London, Canada; Department of Epidemiology, Maastricht University, Maastricht, the Netherlands; Department of Precision Health, Luxembourg Institute of Health, Strassen, Luxembourg; Department of Precision Health, Luxembourg Institute of Health, Strassen, Luxembourg; Living Conditions Department, Luxembourg Institute of Socio-Economic Research, Esch-sur-Alzette, Luxembourg; Living Conditions Department, Luxembourg Institute of Socio-Economic Research, Esch-sur-Alzette, Luxembourg; Department of Precision Health, Luxembourg Institute of Health, Strassen, Luxembourg; Department of Precision Health, Luxembourg Institute of Health, Strassen, Luxembourg; Department of Epidemiology, Maastricht University, Maastricht, the Netherlands; MBP holding, Heerlen, the Netherlands; Department of Precision Health, Luxembourg Institute of Health, Strassen, Luxembourg; Living Conditions Department, Luxembourg Institute of Socio-Economic Research, Esch-sur-Alzette, Luxembourg

**Keywords:** Survey of Health, Ageing and Retirement in Europe (SHARE), Long Covid, multilevel model, multimorbidity, older people, socio-economic factors

## Abstract

**Background:**

A substantial proportion of individuals continue experiencing persistent symptoms following the acute stage of their Covid-19 illness. However, there is a shortage of population-based studies on Long Covid risk factors.

**Objective:**

To estimate the prevalence of Long Covid in the population of middle-aged and older Europeans having contracted Covid-19 and to assess the role of multimorbidity and socio-economic characteristics as potential risk factors of Long Covid.

**Methods:**

A population-based longitudinal prospective study involving a sample of respondents 50 years and older (*n* = 4,004) from 27 countries who participated in the 2020 and 2021 Survey of Health, Ageing and Retirement in Europe (SHARE), in particular the Corona Surveys. Analyses were conducted by a multilevel (random intercept) hurdle negative binomial model.

**Results:**

Overall, 71.6% (95% confidence interval = 70.2–73.0%) of the individuals who contracted Covid-19 had at least one symptom of Long Covid up to 12 months after the infection, with an average of 3.06 (standard deviation = 1.88) symptoms. There were significant cross-country differences in the prevalence of Long Covid and number of symptoms. Higher education and being a man were associated with a lower risk of Long Covid, whilst being employed was associated with a higher risk of having Long Covid. Multimorbidity was associated with a higher number of symptoms and older age was associated with a lower number of symptoms.

**Conclusion:**

Our results provide evidence on the substantial burden of Long Covid in Europe. Individuals who contracted Covid-19 may require long-term support or further medical intervention, putting additional pressure on national health care systems.

## Key Points

- 71.6% of individuals who contracted Covid-19 had at least one symptom of Long Covid up to 12 months after infection.
- Significant cross-country differences in the prevalence of Long Covid and number of symptoms.
- Higher education and being a man were associated with a lower risk of Long Covid, being employed with a higher risk.
- Multimorbidity was associated with a higher number of symptoms, employed and older age were associated with a lower number.

## Background

The coronavirus disease (Covid-19) pandemic has resulted in ˃676 million confirmed cases and over 6.8 million deaths worldwide [1]. Over 40% of individuals who contracted Covid-19 continue to report symptoms or develop new symptoms following the acute stage of their SARS-CoV-2 virus infection [2]; that is, they experience post-acute Covid-19, commonly referred to as Long Covid [3]. The World Health Organization defines Long Covid as a condition that occurs in individuals with probable or confirmed Covid-19, 3 months from the onset of infection with symptoms that last for at least 2 months [4].

The severity of the acute stage of the Covid-19 infection and being a woman are the most consistently reported risk factors for Long Covid [2, 5]. Pre-existing chronic conditions such as lung diseases [2, 6], mental health disorders [7] and the number of co-existing chronic diseases [8] may also increase the risk of Long Covid. As with other diseases, socio-economic factors play an important role in the Covid-19 pandemic, with socio-economically disadvantaged groups and geographic areas most heavily affected [9–12]. Emerging evidence on the effects of socio-economic factors such as education [6] and income [7, 13, 14] on Long Covid is limited, highlighting the need for more empirical assessments with large representative community samples.

The objective of this study was to estimate the prevalence of Long Covid in the population of middle-aged and older adults (≥50 years old) in Europe that have contracted Covid-19 and to investigate the role of multimorbidity and socio-economic factors (i.e. immigration status, education, employment status and household income) as potential correlates of Long Covid. We also assessed how the prevalence of Long Covid varied across the 27 European countries participating in the Survey of Health, Ageing and Retirement in Europe (SHARE) [15].

## Methods and materials

### Study population

We used data from the 2020 SHARE Corona Survey 1 [16] and 2021 Corona Survey 2 [17] that were collected in response to the Covid-19 pandemic across Europe (*n* = 47,964). These two surveys offer a rare opportunity to follow a large cohort of middle-aged and older adults over a 1-year period to assess the impact of Covid-19 in this population. As illustrated in Supplementary Figure 1, our sample consisted of 4,004 respondents aged 50 years and older who, at the Corona Survey 2 (summer 2021), indicated that, since the Corona Survey 1 (summer 2020), they either (i) had a positive test for the SARS-CoV-2 virus (*n* = 3,103) or (ii) experienced symptoms that they attributed to the Covid-19 illness but were not tested (*n* = 901).

### Long Covid

Respondents were asked ‘Have you experienced at least one long-term or lingering effect that you attribute to your Covid illness?’ and were provided with a list of nine symptoms: fatigue; cough, congestion, shortness of breath; loss of taste or smell; headache; body aches, joint pain; chest or abdominal pain; diarrhoea, nausea; confusion or any other symptoms. We created a count variable ranging from 0 (no symptoms) to 9 (the maximum number of symptoms). In line with previous meta-analyses [3, 18] and population-based studies [13, 19–21], respondents were considered having Long Covid, if they reported one or more of these symptoms during the 12-month follow-up.

### Covariates of Long Covid

We defined multimorbidity as a self-reported diagnosis of two or more of the following seven chronic conditions: hip fracture; diabetes or high blood sugar; hypertension; heart attack or other heart problem; chronic lung disease; cancer or malignant tumour; and other illness or health condition [22, 23]. Information on multimorbidity was collected in the Corona Survey 1 or in previous SHARE surveys, depending on when the respondents first reported these conditions. We categorised respondents as ‘non-immigrants’ and ‘immigrants’, depending on whether or not their country of birth was the same as the country of interview. We constructed two categories of education: ‘secondary or lower’ and ‘post-secondary or higher’. Employment (‘employed’ versus ‘not employed’) and household income (the lowest monthly household income since the Covid-19 outbreak) were derived from the Corona Survey 1. Income was adjusted by household size and converted into quintiles.

### Data analysis

To assess the impact of the correlates on the risk of Long Covid (and on the number of symptoms), we used a multilevel (random intercept) hurdle negative binomial model (truncated at zero) with a mixture of two distributions: a binary model for the risk of Long Covid (reporting at least one symptom) and a count model for the number of symptoms [24, 25]. In a preliminary analysis, we ran a series of univariate models to assess each correlate (i.e. multimorbidity, immigration, education, employment and income) individually. These results (see Supplementary Table 1) suggested that immigration and income were not significantly associated with the risk of Long Covid or with the number of symptoms. To assess the independent effects of all correlates, we carried out a multivariable hurdle model. For the fixed effects, we computed odds ratios (OR) and rate ratios (RR) for the risk of Long Covid and the number of symptoms, respectively. To assess cross-country differences, we used the variances and a covariance of the two random intercepts (τ^2^_binary_, τ^2^_count_, τ^2^_cov_). In all models, we controlled for age (in years) and gender. We centred the correlates using their grand means and proportions [26]. The standard errors (SEs) were computed using restricted maximum-likelihood and a *P*-value of 0.05 was used in all tests. There were 186 (4.7%) respondents with missing data points, including 13 (0.3%) for the outcome variable. We used full-information maximum likelihood to model the missingness under the missing at random assumption [27] and we applied calibrated cross-sectional sampling weights from the Corona Survey 2. We used SAS 9.4 [28] and Mplus [29] to conduct the analysis.

## Results

Of the 4,004 respondents who had Covid-19 (average age 64.6 years; 55.8% women), 71.6% (95% CI = 70.2–73.0%) (*n* = 2,857/3,992) experienced Long Covid with an average of 3.06 (SD = 1.88) symptoms. Fatigue (50.1%), cough, congestion, shortness of breath (36.0%) and body aches (30.9%) were the three most prevalent symptoms. Table 1 shows the unweighted frequencies and weighted percentages for symptoms of Long Covid whilst Table 2 provides proportions and means for prevalence of Long Covid and the number of symptoms across the correlates and for each of the 27 countries.

**Table 1:** Descriptive statistics for symptoms of Long Covid

**Variable**	**Category**	**Unweighted Frequencies**	**Weighted Percentages** ^**a**^
Long-term	Fatigue	2,007	50.12
symptoms	Cough/congestion/shortness of breath	1,441	35.99
	Loss of taste/smell	1,237	30.89
	Headache	1,133	28.30
	Body aches/joint pain	1,305	32.59
	Chest/abdominal pain	580	14.49
	Diarrhoea/nausea	394	9.84
	Confusion	320	7.99
	Other symptoms	626	15.63
	No Symptoms	1,118	28.40
Number of	No symptoms	1,118	28.35
symptoms	1 symptom	656	18.94
	2 symptoms	607	14.00
	3 symptoms	556	12.87
	4 symptoms	379	9.52
	5 symptoms	295	6.85
	6 symptoms	217	5.16
	7 symptoms	102	2.38
	8 symptoms	51	1.47
	9 symptoms	10	0.15
	Missing	13	0.31

Source: SHARE, Corona Survey 2 (summer 2021).

*n* = 4,004 respondents from 27 countries.

^a^Calibrated individual sampling weights from the Corona Survey 2 were used.

**Table 2:** Descriptive statistics for the presence of Long Covid and the number of symptoms by covariates

**Variable**	**Category**	**Unweighted Frequencies**	**Weighted Percentages** ^**a**^	**Proportion of Individuals with Long Covid**	**Average Number of Symptoms** ^**b**^
Multimorbidity	Multimorbid	1,415	29.27	0.73	3.26
	Not multimorbid	2,506	68.25	0.71	2.96
	Missing	83	2.48		
Immigration	Immigrants	349	8.55	0.77	3.03
status	Non-immigrants	3,576	89.02	0.71	3.26
	Missing	79	2.43		
Educational	Higher level	1,135	31.30	0.66	3.01
attainment	Lower level	2,783	66.18	0.74	3.08
	Missing	86	2.53		
Employment	Employed	1,267	50.53	0.73	2.96
status	Not employed	2,719	49.01	0.70	3.17
	Missing	18	0.46		
Household	1st quintile	769	19.20	0.73	3.18
income	2nd quintile	800	18.04	0.73	3.05
	3rd quintile	804	19.51	0.73	2.95
	4th quintile	780	18.53	0.69	3.18
	5th quintile	764	22.15	0.71	3,04
	Missing	87	2,57		
Country of	Austria	116	2,90	0,73	2,13
residence	Germany	62	1.55	0.40	2.71
	Sweden	91	2.27	0.47	2.26
	Netherlands	55	1.37	0.43	2.02
	Spain	130	3.25	0.48	2.30
	Italy	198	4.95	0.74	4.10
	France	98	2.45	0.56	2.56
	Denmark	61	1.52	0.58	2.86
	Greece	113	2.82	0.66	3.40
	Switzerland	114	2.85	0.76	1.41
	Belgium	289	7.22	0.69	3.17
	Czech Republic	315	7.87	0.84	2.41
	Poland	453	11.31	0.86	3.74
	Luxembourg	85	2.12	0.63	2.73
	Hungary	74	1.85	0.89	5.04
	Portugal	53	1.32	0.66	1.96
	Slovenia	401	10.01	0.66	3.14
	Estonia	357	8.92	0.80	3.29
	Croatia	204	5.09	0.89	3.33
	Lithuania	141	3.52	0.75	2.33
	Bulgaria	131	3.27	0.81	3.09
	Cyprus	43	1.07	0.78	3.19
	Finland	84	2.10	0.28	2.59
	Latvia	68	1.70	0.80	2.78
	Malta	55	1.37	0.38	2.17
	Romania	120	3.00	0.64	2.92
	Slovakia	93	2.32	0.75	3.20
Gender	Woman	2,363	55.79	0.75	3.11
	Man	1,641	44.21	0.68	3.00
Age	50–64	1,465	59.92	0.72	3.09
	65–79	2,066	31.99	0.71	3.07
	80 plus	473	8.09	0.71	2.87

Source: SHARE, Corona Survey 1 (summer 2020) and Corona Survey 2 (summer 2021), *n* = 4,004 respondents from 27 countries.

^a^Calibrated individual sampling weights from the Corona Survey 2 were used.

^b^Average number of symptoms related to Long Covid for individuals with one or more symptoms (i.e. those who had Long Covid).

The parameter estimates for the fixed and random effects with their SEs, *P*-values, and 95% CIs from the multivariable hurdle model are presented in Table 3. The results from the binary part of the model indicate that higher education (OR = 0.72; 95% CI = 0.52–0.99) and being a man (OR = 0.69, 95% CI = 0.52–0.92) were significantly associated with a lower risk of Long Covid whilst employment (OR = 1.53; 95% CI = 1.18–1.98) was significantly associated with a higher risk of Long Covid. Specifically, respondents with post-secondary or higher levels of education were 28% less likely to have Long Covid than those with secondary or lower levels of education. Men were 31% less likely than women to have Long Covid. Respondents who were employed at the time of the Corona Survey 1 (i.e. before they contracted Covid-19) were 53% more likely to have Long Covid than those who did not work at that time. Age, multimorbidity, immigration status and income did not have a statistically significant impact on the risk of Long Covid. We also found that multimorbidity (RR=1.12; 95% CI = 1.02–1.22) and age (RR = 0.92; 95% CI = 0.86–0.98) were significantly related to the number of symptoms associated with Long Covid. Respondents with multimorbidity had a 12% increased risk of experiencing an additional symptom whilst older respondents experienced, on average, fewer symptoms. There was no significant association between gender, immigration, education and income with the number of symptoms.

**Table 3:** Fixed and random effects from the multivariable multilevel hurdle model

**Component**	**Fixed Effects**	**Estimate**	**SE**	***P*-value**	**OR/RR**	**Lower CI**	**Upper CI**
Binary model	Intercept	1.57	0.75	0.04	4.78	1.10	20.78
	Gender (man)	–0.37	0.15	0.01	0.69	0.52	0.92
	Age (average)	0.16	0.08	0.06	1.17	0.99	1.38
	Multimorbidity (multimorbid)	0.09	0.14	0.54	1.09	0.83	1.44
	Immigration (immigrant)	0.41	0.28	0.14	1.51	0.87	2.59
	Education (higher)	–0.33	0.17	0.05	0.72	0.52	0.99
	Employment (employed)	0.42	0.13	0.00	1.53	1.18	1.98
	Income (1st quintile)	–0.06	0.25	0.80	0.94	0.58	1.53
	Income (2nd quintile)	–0.10	0.28	0.72	0.90	0.52	1.57
	Income (4th quintile)	–0.43	0.28	0.13	0.65	0.38	1.13
	income (5th quintile	–0.24	0.26	0.34	0.79	0.48	1.30
Count model	Intercept	1.06	0.10	0.00	2.89	2.38	3.51
	Gender (man)	–0.04	0.05	0.47	0.97	0.88	1.06
	Age (average)	–0.09	0.03	0.01	0.92	0.86	0.98
	Multimorbidity (multimorbid)	0.11	0.05	0.01	1.12	1.02	1.22
	Immigration (immigrant)	0.12	0.08	0.15	1.13	0.96	1.33
	Education (higher)	0.02	0.07	0.76	1.02	0.89	1.17
	Employment (employed)	–0.16	0.08	0.06	0.85	0.72	1.01
	Income (1st quintile)	0.06	0.08	0.46	1.06	0.90	1.25
	Income (2nd quintile)	–0.01	0.07	0.92	0.99	0.86	1.15
	Income (4th quintile)	0.04	0.07	0.55	1.04	0.91	1.20
	Income (5th quintile	0.00	0.07	0.97	1.00	0.87	1.15
	Dispersion	0.11	0.05	0.02		0.02	0.20
**Model**	**Random Effects**	**Variance**	**SE**	***P*-value**		**Lower CI**	**Upper CI**
Binary model	Intercept (τ2binary)	0.60	0.15	0.00		0.30	0.90
Count model	Intercept (τ2count)	0.11	0.05	0.04		0.01	0.21
	Covariance (τ2cov)	0.11	0.04	0.02		0.02	0.19

Source: SHARE, Corona Survey 1 (summer 2020) and Corona Survey 2 (summer 2021), *n* = 4,004 respondents from 27 countries.

Binary model: a logistic component of the multilevel hurdle model to estimate the odd ratios for the presence of Long Covid Count model: a Poisson component of the hurdle multilevel model with negative binomial distribution to estimate the rate ratios for the number of symptoms related to Long Covid.

OR: odds ratio; RR: rate ratio; SE: standard error; CI: confidence interval; τ2: variance for the random effect. Chi-squared = –23,833; AIC = 47,745; BIC = 47,997.

**Figure 1:**
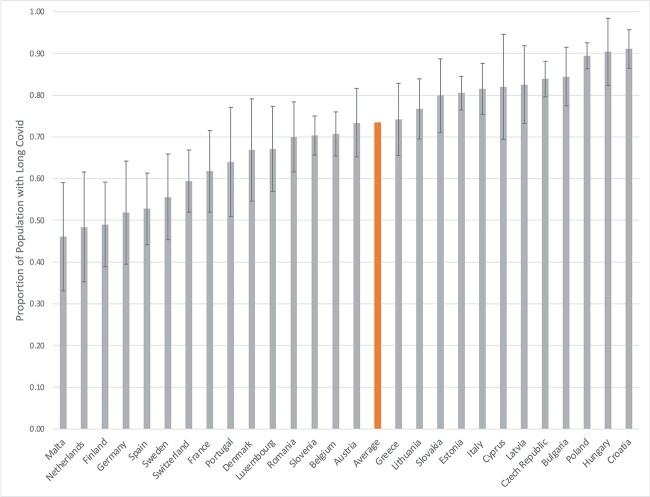
Estimated proportion of individuals with Long Covid by country of residence (with 95% confidence intervals).

**Figure 2:**
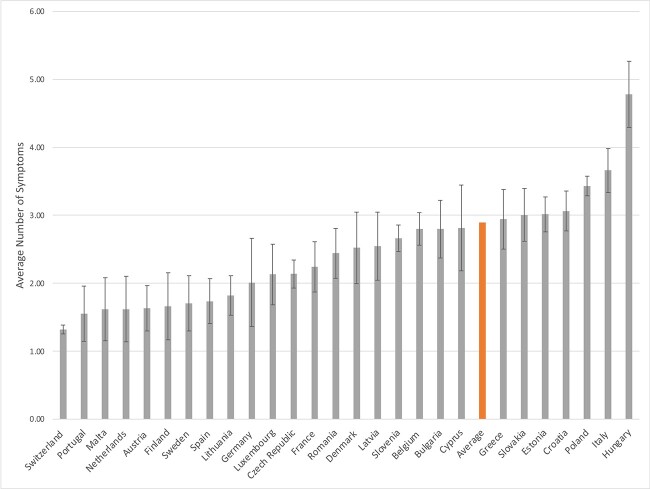
Estimated average number of symptoms related of Long Covid by country of residence (with 95% confidence intervals).

The variances for the intercepts in the binary and count parts of the model (τ^2^_binary_ = 0.60, 95% CI = 0.30–0.90; τ^2^_count_=0.11, 95% CI = 0.01–0.21) were statistically significant, suggesting that, controlling for all the correlates, there were significant cross-country differences in the proportion of the individuals having Long Covid and in the average number of symptoms. The estimated proportion of the individuals who had Long Covid was the highest in Croatia (91%) and lowest in Malta (46%); the estimated counts of symptoms per person varied across countries from 1.23 in Switzerland to 4.78 in Hungary (see Figures 1 and 2, respectively, and Supplementary Table 2). There was also a statistically significant positive covariance between the two random intercepts (τ^2^_cov_ = 0.11; 95% CI = 0.02–0.19), suggesting that countries with a higher proportion of individuals having Long Covid also had a higher average number of symptoms.

## Discussion

To the best of our knowledge, SHARE’s Corona Survey is one of the largest population-based longitudinal prospective studies, including representative samples from 27 European countries. With the sampling weights and inclusion of individuals with self-diagnosed Covid-19 infections, these data allowed us to produce valid, reliable and realistic prevalence estimates of Long Covid in the general population of middle-aged and older adults who contracted Covid-19. An important finding of the current study is that, during the summer of 2021, seven out of ten middle-aged and older Europeans reported having long-term symptoms attributable to Covid-19 illness, up to 12 months post-infection by the SARS-CoV-2 virus. This estimate is within the confidence intervals of the pooled prevalence estimates of 64% (95% CI, 54–74%) at 6 months and 59% (95% CI, 46–72%) at 12 months post-infection, reported in a meta-analysis of European studies [18]. However, our estimate is slightly higher than the pooled prevalence estimate for European counties reported in a Lancet meta-analysis (63%; 95% CI, 57–69%), though the average days of follow-up in that study was only 126 and it included individuals of all ages [3]. Within the context of other population-based studies, our country-specific prevalence estimate for Germany (44%; 95% CI, 32–57%), for instance, is lower than an estimate of 64% at 6–12 months for southern Germany [19]. We also found that participants in the Corona Survey 2 reported, on average, three out of the possible nine symptoms. It is challenging to contextualise this finding as most comparable studies included a much larger number of symptoms in their questionnaires (up to 30) [13, 19, 21]. In line with previous research, we found that fatigue was the most often reported symptom [2, 3, 18].

Our study is the first to compare the prevalence of Long Covid across a large number of countries using survey data collected with a standardised sampling design and survey questionnaire. We also adjusted our population-based estimates for important individual-level socio-demographic and health characteristics. Most of the previous studies on Long Covid are country-specific, followed different study populations (e.g. hospitalised versus non-hospitalised), adopted different sample designs (e.g. non-probability versus representative) or had different follow-up periods. All these factors contribute to a substantial level of heterogeneity across estimates reported in other studies, meaning their results are not easily comparable with ours. For instance, a recent meta-analysis shows that the prevalence estimates from individual European studies ranged from 9% to 81% [2].

Our study also found substantial cross-country differences in the proportion of individuals having Long Covid, with country-specific estimates ranging from 46% in Malta to 91% in Croatia. The novel and unique methodological features of our study gave us a high level of confidence that these differences reflect the true cross-country differences in the prevalence of Long Covid, as national samples were collected using the same methodology. We also found similarly substantial cross-country differences in the average number of symptoms and found that countries with a higher prevalence of Long Covid also had a larger average number of symptoms. We hypothesise that this substantial variation can be partially attributable to differences in availability and access to testing, as well as media attention surrounding Long Covid and its symptoms [21]. However, it may also reflect some broad cultural and environmental differences across European countries [30]. Future comparative research should be undertaken to investigate these and other potential factors to explain the cross-country differences.

Another objective of this study was to investigate the role of multimorbidity and socio-economic characteristics as potential risk factors for Long Covid as there is a paucity of population-based studies in this area. The longitudinal prospective design of the SHARE data permitted us to measure correlates of Long Covid (i.e. multimorbidity, employment and income) 1 year prior to respondents’ reporting of their symptoms of Long Covid. In terms of our findings, although multimorbidity was not correlated with the risk of Long Covid, it had a statistically significant impact on the number of symptoms. These results are consistent with a Spanish study involving hospitalised patients [8] and suggest that individuals diagnosed with multiple chronic conditions were not at a higher risk of being affected by Long Covid but those who contracted Covid-19 were more likely to experience a larger number of persistent symptoms commonly attributed to Covid-19 illness. These results imply that individuals living with multimorbidity have different recovery trajectories than those without multimorbidity.

We found that respondents with post-secondary or higher levels of education were 28% less likely to have Long Covid, which suggests that these individuals were in a better position to take advantage of some of the available measures to protect themselves against severe outcomes of Covid-19 illness. Education, however, had no significant effect on the number of symptoms. These results were inconsistent with findings from a large population-based study from the UK [6] and a smaller Canadian study involving patients discharged from hospital [20]. Respondents who were employed before their Covid-19 illness were 53% more likely to have Long Covid. Since we did not find any comparable studies and we do not have data on whether respondents were still working during or after the period of their illness or on their employment conditions (e.g. work from home, usage of public transportation, access to adequate protection), it is difficult to speculate about the causal mechanism linking employment with Long Covid. Although we did not find household income and immigration status to be correlated with Long Covid, in population-based studies conducted in the UK and US, individuals from low-income households were found to be at a higher risk of Long Covid [7, 13, 14]. Finally, consistent with the results from the recent meta-analysis [2], we found that women were more likely than men to have Long Covid but did not experience a larger number of symptoms. In line with some evidence that older adults are more likely to be asymptomatic [13], we found that older respondents who had Long Covid reported, on average, fewer symptoms; older adults were also not at a higher risk of Long Covid. Currently, there is no consensus on the relationship between age and Long Covid, with some studies pointing to a U-shaped relationship [6].

Although our results suggest that individuals who are employed, those with lower levels of education, and women might be at a higher risk of Long Covid, there may be other factors that should be investigated in future research, including health-related behaviours, psychological factors, social support or the characteristics of local public health systems. Identification of risk factors for Long Covid would allow policymakers and health care practitioners to focus their efforts on addressing the needs of sub-populations of adults that are the most susceptible to the long-term effects of Covid-19 illness. More research however is needed in this area.

Our study has several limitations. Our estimates are assumed to reflect the prevalence of Long Covid ‘up to 12 months’ since the onset of the infection. In the Corona Survey 2, respondents reported long-term symptoms of Covid-19 that occurred at any time after the Corona Survey 1. However, they were not asked to provide information on the date of infection or the duration of these symptoms. Thus, although our assumption of the duration of the follow-up seems to be reasonable, in some cases, the reported symptoms may reflect symptoms that were present during the acute phases of the illness or started before the 3-month interval. Thus, it is possible that our estimates overestimate the true prevalence of Long Covid. As in other population-based surveys, we used self-reported data and respondents were not asked whether their symptoms were severe, which may contribute to the self-report bias and higher prevalence estimates. Another limitation is the possibility of recall bias since respondents were asked to report their symptoms up to 1 year after Covid-19 onset. Finally, there is some emerging evidence that symptoms commonly associated with Long Covid should not be considered as a single condition, but rather as multiple clusters of symptoms [13, 19, 31].

## Conclusions

This is one of the largest population-based longitudinal studies in Europe and it provides strong evidence on the substantial burden of Long Covid. We found that seven in ten middle-aged and older adults had persistent symptoms of Covid-19 illness, up to 12 months since the infection. We also found significant cross-country differences in the prevalence of Long Covid and number of symptoms. In terms of correlates of Long Covid, higher education and being a man were associated with a lower risk of Long Covid, whilst being employed was associated with a higher risk. In addition, multimorbidity status was associated with a higher number of symptoms, whilst older age was associated with a lower number of symptoms.

Symptoms of Long Covid reported in the literature were found to be related with a poorer quality of life, decline in mental health and well-being and stress [18, 21, 32]. Thus, many individuals who are affected by this disease may require long-lasting support or further medical intervention, putting additional pressures on national health care systems [13]. To address these challenges, there is a need for longitudinal studies to monitor the health trajectories of individuals living with Long Covid and to identify groups of individuals at highest risk. There is also a need for national comprehensive surveillance programmes to inform policymakers, healthcare professionals and other stakeholders to better manage future health care and rehabilitation needs of patients affected by the current and any future pandemics [18].

## Supplementary Material

aa-23-0335-File002_afad225
