# Supplementary material for: The role of multimorbidity and socio-economic characteristics as potential risk factors for Long Covid: evidence from the multilevel analysis of the Survey of Health, Ageing and Retirement in Europe’s corona surveys (2020–2021)
Source: Age Ageing. 2023 Dec 19;52(12):afad225. doi: 10.1093/ageing/afad225 (PMC10733586; doi:10.1093/ageing/afad225)
Supplement: aa-23-0335-File002_afad225 [file aa-23-0335-file002_afad225.docx]

**The Role of Multimorbidity and Socio-economic Characteristics as Potential Risk Factors for Long Covid: Evidence from the Multilevel Analysis of the Survey of Health, Ageing and Retirement in Europe’s Corona Surveys (2020-2021)**

**Supplementary Files**

**Supplementary figure 1.** Study sample flow diagram

**Supplementary table 1.** Additional models tested.

**Supplementary Table 2.** Additional multivariable models.
